# Chinese version of the Global Youth Tobacco Survey: cross-cultural instrument adaptation

**DOI:** 10.1186/1471-2458-8-144

**Published:** 2008-04-30

**Authors:** Ping-Ling Chen, Hung-Yi Chiou, Yi-Hua Chen

**Affiliations:** 1 School of Nursing, Taipei Medical University, Taipei, Taiwan; 2 School of Public Health, Taipei Medical University, Taipei, Taiwan

## Abstract

**Background:**

Tobacco smoking poses public health concerns because of its high risk for many chronic diseases. Most smokers begin using tobacco in their teens and recent reports indicate that smoking prevalence is climbing among youth. The Global Youth Tobacco Survey (GYTS) is a worldwide, school-based, tobacco-specific survey, but cross-cultural differences limit its effectiveness in international studies. Specifically, the GYTS assesses not only the prevalence of smoking, but also tobacco-related attitudes, school curricula, and advertisements, which are culturally influenced. Therefore, we conducted this study to develop a Chinese version of the GYTS for both national surveillance and international comparison.

**Methods:**

The original English GYTS was translated and back translated using a cross-cultural adaptation process. The comprehensiveness and feasibility of using the Chinese-version GYTS were reviewed by a panel of 6 tobacco-control experts. The understandability and cultural relevance of the Chinese-version GYTS were discussed in a focus group of 5 schoolteachers and 8 students. The expert and focus group feedback was incorporated into a final Chinese version of the GYTS, which was administered to 382 students throughout Taiwan by multi-stage sampling from 10 randomly selected schools.

**Results:**

The internal consistency (Cronbach's alpha) for the GYTS subscales (smoking susceptibility, attitude toward smoking, and media messages about smoking) ranged from 0.70 to 0.94. The internal logical agreement of responses ranged from 85.3 to 99.2%.

**Conclusion:**

The Chinese version of the GYTS has good reliability and validity and can serve as the foundation for international comparison and tobacco control in Chinese-speaking communities.

## Background

Tobacco use is a major preventable cause of premature morbidity and mortality among men and women [1,2]. To address this issue, the World Health Organization (WHO) Framework Convention on Tobacco Control urged its member nations to install national smoking surveillance systems [3]. These systems would serve as a basic infrastructure to understand the effects of activities that promote tobacco use, identify high-risk groups and service needs, build support for comprehensive policies and programs, evaluate tobacco control interventions, make cross-country comparisons, and direct future tobacco-related research [3].

Due to a trend of shifting the geography of smoking from developed to developing countries, almost half (46.8%) of adult men and 4% of adult women in Taiwan are smokers, resulting in over 18,000 deaths from tobacco-related diseases each year [4]. Tobacco control in Taiwan faced several challenges in 1987, as in other Asian countries, due to opening the tobacco market to foreign companies. For instance, during the first 5 years after deregulation, aggressive foreign cigarette marketing strategies were associated with a 13% increase in the prevalence of smoking by young adult males, and the market share of foreign cigarettes among young smokers grew to exceed domestic cigarettes by 3 to 1 [5]. Furthermore, this increased tobacco marketing directed at young people resulted in their initiating smoking at a lower age [6].

Since most smokers begin using tobacco in their teens, a youth tobacco-surveillance program needed to be established in Taiwan. Such a program needed to be evidence-based, but previous surveys on tobacco use in Taiwan were not comparable to each other or to data from other countries because they used several survey instruments, sampling frames, and data collection protocols [7,8]. To address this deficiency in tobacco use data, the Taiwan Bureau of Health Promotion implemented the Global Youth Tobacco Survey (GYTS) with the cooperation of the US Centers for Disease Control and Prevention (CDC). The GYTS, part of the Global Tobacco Surveillance System (GTSS) initiated by the WHO, CDC, and the Canadian Public Health Association, was developed to monitor tobacco use, attitudes, curricula, and exposure to tobacco advertising and environmental tobacco smoke among adolescents aged 13–15 years. These outcomes were assessed using a standardized methodology for constructing the sample frame, selecting schools and classes, preparing uniform questionnaires, and following consistent field procedures [9]. Since tobacco-related attitudes, curricula, and advertisements in items of the GYTS are culture-oriented, we conducted this study to adapt the original English-version GYTS to Chinese for both national surveillance and international comparison.

## Methods

### Global Youth Tobacco Survey

The GYTS is a self-administered, school-based instrument consisting of 56 core questions designed to gather data on the following 7 domains: prevalence of cigarette and other tobacco use, perceptions and attitudes about tobacco, access to and availability of tobacco products, exposure to secondhand smoke, school curricula, media and advertising, and smoking cessation [10]. The GYTS uses a generic answer sheet with 8 response categories available per question. The numbers of response categories are varied by the nature of questions. There are no open-ended questions, no skip patterns, and no multiple response questions in the GYTS. To ensure the cultural appropriateness and equivalence of the Chinese and English versions of the GYTS, a cross-cultural adaptation process is recommended by Guillemin et al. [11]. This process, which includes forward translation, expert committee review, focus group discussion, back translation, and a field test, was applied in this study to modify the original, validated English version for use with Chinese-speaking youth.

### Stage 1: Forward translation

The forward translation was independently performed by 2 bilingual epidemiologists. Before translating the GYTS, they participated in a 2-day GYTS workshop held by the US CDC to familiarize them with the detailed concepts of the GYTS. After translating the GYTS, the epidemiologists discussed their translations and reached agreement to generate the first Chinese version of the GYTS.

### Stage 2: Expert committee

The appropriateness and comprehensiveness of the Chinese-version GYTS was reviewed by an expert panel who identified and fully discussed translation discrepancies with the source-version GYTS and cultural barriers. Besides the 3 researchers, the panel included 6 epidemiologists with expertise in risk-behavior surveillance and tobacco control. These epidemiologists were 5 university professors from 4 medical universities and 1 government employee from the Taiwan Bureau of Health Promotion. By consensus of these experts, the second version of the Chinese GYTS was developed.

### Stage 3: Focus group

Since the Chinese-version GYTS was designed to survey smoking behavior among Taiwanese teenagers who are junior and senior high school students, the second version of the translated questionnaire was discussed in a focus group of 5 health-education teachers and 8 students from 2 junior high schools, 1 senior high, and 1 vocational high in Taipei. The focus group was led by the first author. The participants included 3 female teachers from 3 junior highs, 1 female teacher from a senior high, 1 male teacher from a vocational high, 2 male and 2 female junior high students, and 2 male and 2 female senior (vocational) high students. The Chinese-version GYTS was distributed, and participants were asked to take 20 minutes to review and answer the questionnaire. They were then asked to give their opinions about the clarity of survey items and whether these items reflect the current situation of teenagers regarding the prevalence of cigarette and other tobacco use, perceptions and attitudes about tobacco, access to and availability of tobacco products, exposure to secondhand smoke, school curricula, media and advertising, and smoking cessation. Through abundant discussion, the focus group participants clarified response options of items related to cigarette brand preference, attitudes toward cigarette smoking, and cultural tobacco issues. The whole discussion was tape-recorded, and the tape was transcribed verbatim. The transcript identified participants only by code number and was used as the foundation to modify the questionnaire.

### Stage 4: Back translation

After the focus group, the third Chinese version of the GYTS was translated back into English by one university English instructor for equivalence assessment. This back-translated GYTS was compared to the original English version by 2 other university English professors to ensure that two versions had the same meaning. Each item was independently scored for equivalence on a 5-point scale (1 = "very different," 5 = "very similar").

### Stage 5: Field test

To evaluate the applicability and cultural acceptability of the third Chinese version of the GYTS, it was treated as a new instrument and field tested in June, 2004. Representative students were sampled using a multi-stage cluster sample design. First, one county was randomly selected from the northern, central, southern, and eastern areas of Taiwan. Second, one junior and one senior high school were randomly chosen from each selected county. Because the junior and senior high schools selected in the southern area were all-girl schools, another junior and another senior high school were randomly selected from that area. Third, one class was randomly selected from each selected school. Because twelfth-grade students were preparing for the national college entrance examination at the time of sampling, this group of students was excluded in the sampling frame. All students in the selected classes were invited to participate in the field test.

The sample size of the field test was 412 students, from whom 382 usable questionnaires were collected, for a response rate of 92.7%. The field test sample (n = 382) included 44.8% male and 55.2% female respondents from age 12 to 17 years, seventh to eleventh graders. The field test results showed that 36.13% of participants had tried smoking even 1 or 2 puffs, and 11.78% were current smokers.

The test-retest reliability of the third Chinese-version GYTS was evaluated by administering the same questionnaire 2 weeks after the field test to 2 classes at 2 randomly selected schools (i.e., 20% of the total sample). The participants in this second field test included 50 tenth graders and 40 eighth graders. From these 90 students, 89 usable questionnaires were collected, for a response rate of 98.9%. All questionnaires were collected using a standardized procedure developed by the GTSS [12]. After the field test, the final Chinese-version GYTS [see Additional file 1] was back translated to English and mailed to the US CDC to obtain approval of the original authors.

### Ethical Considerations

Before the focus group, all student and teacher participants signed consent forms. The confidentiality of their verbatim-transcribed statements was ensured by using code numbers for each participant. For the field test, formal invitation letters were mailed to principals of all participating schools, and they gave verbal consent. Passive consent was obtained from every student by delivering parent-notification letters to their parents before data collection. Student respondents' confidentiality was ensured by anonymous self-administration of questionnaires.

### Data Analysis

The content validity and face validity of the Chinese-version GYTS were evaluated by the cross-cultural adaptation process and by qualitative analyses of participants' comments in the expert committee and focus group. The mean equivalence scores for items in the back-translated and original English versions of the GYTS were analyzed by descriptive statistics. The reliability of the Chinese-version GYTS was determined by Cronbach's alpha and percent agreement due to the nature of the subscale scoring system. Because GYTS surveys were completed anonymously, individual data could not be linked between the test and retest. Therefore, differences in the overall distribution of subscale scores were compared by t-test. All data analyses were run using SAS, version 8.0, statistical software (SAS Institute, Inc., Cary, North Carolina).

## Results

### Forward translation, expert review, and focus group

In the first stage of the cross-cultural instrument development process, the GYTS was forward translated without any major problem because the translators had fully discussed the GYTS with a major contributor to the GTSS Collaborating Group during their 2-day GYTS workshop. In the second and third stages, expert and focus group review, some minor semantic and idiomatic discrepancies were discussed at length. Most of these discrepancies were related to response options for smoking-related attitudes. As recommended by the experts and focus group participants, GYTS items regarding tobacco price, monthly tobacco expenditures, and monthly allowance were modified to fit consumer spending in Taiwan. Options for cigarette brands were also modified since brand preference is associated with the local culture and marketing strategies used in Taiwan. Survey items related to other forms of tobacco were revised to include only chewing tobacco, cigars, and pipe, thus reflecting that Taiwanese smokers mainly consume cigarettes. The response category related to cigarette-vending machines was deleted from items related to tobacco access since Taiwan does not have these machines.

Cultural barriers were found between the original and translated versions of the GYTS. For example, Taiwanese teenagers had difficulty distinguishing between the semantics of the response categories, "social events" and "public places." Therefore, the term "social events" was replaced by "gatherings with friends." Culture-specific items about attitudes toward tobacco were also modified. For example, the item "What would you think of a man when you see him smoking?" has 4 positive response options ("successful," "intelligent," "macho," and "dashing") and 4 negative response options ("lacks confidence," "stupid," "careless," and "loser"). These response options were modified by replacing "macho" with "elegant." The above modifications were primarily made to reflect that Taiwanese teenagers are affected by culture and habits of tobacco use.

### Equivalence of back-translated and original English versions

The Chinese-version GYTS was back translated to English and its items were compared and ranked for equivalence with items on the original English-version GYTS. This item ranking by 2 English assistant professors showed that more than 53% of the average equivalence scores were above 4.5 (Table 1), indicating good item content and cultural equivalence of the source and translated versions.

**Table 1:** Equivalence of items on back-translated and original English versions of GYTS

Mean Equivalence Score^a^	Items	
	
	Number	Percentage
5.0	1	1.8
4.5	29	51.8
4.0	26	46.4
Total	56	100.0

^a ^5 indicates "very similar," 1 indicates "very different"

### Internal consistency of the Chinese-version GYTS

To ensure anonymity of students' smoking status, a "no-skip" design was used in the GYTS. Every item includes the response option, "I have never smoked cigarettes," thus ensuring that every student, regardless of smoking status, answers each item and takes the same time to complete the questionnaire. This "no-skip" design also provided an excellent opportunity to assess the consistency of responses about tobacco-use behavior by comparing the answer to item 1 (Have you ever tried cigarette smoking, even 1 or 2 puffs?) with answers to comparable items (items 2, 35, 36, 37, 38, 39, and 40). For example, if the response to item 1 was "no," but the response to item 2 (How old were you when you first tried a cigarette?) was other than "I have never smoked cigarettes," the response was treated as inconsistent. The consistency of responses, or percent agreement between responses to item 1 and to related items, was 86.7–99.2% (Table 2).

**Table 2:** Consistency of responses to item 1 and other smoking-related items in Chinese-version GYTS (*N *= 382)

Item number	Item content	Agreement (%)
1	Have you ever tried cigarette smoking, even 1 or 2 puffs?	100.0
2	How old were you when you first tried a cigarette?	99.2
35	Do you want to stop smoking now?	90.3
36	During the past year, have you ever tried to stop smoking cigarettes?	89.5
37	How long ago did you stop smoking?	87.4
38	What was the main reason you decided to stop smoking?	88.0
39	Do you think you would be able to stop smoking if you wanted to?	86.7
40	Have you ever received help or advice to help you stop smoking?	88.2

The consistency of responses about tobacco-use behavior was also assessed using another item related to smoking frequency, item 3 (During the past month, on how many days did you smoke cigarettes?). The response to item 3 was compared to responses to other smoking-related items (items 4, 5, 6, 7, 8, 12, and 13). The agreement among responses to these items was high, 85.3–99% (Table 3), demonstrating good internal consistency of the Chinese-version GYTS.

**Table 3:** Consistency of responses to item 3 (smoking frequency) and to related items in the Chinese-version GYTS (*N *= 382)

Item number	Item content	Agreement (%)
3	During the past 30 days (one month), on how many days did you smoke cigarettes?	100.0
4	During the past 30 days (one month), on the days you smoked, how many cigarettes did you usually smoke?	99.0
5	During the past 30 days (one month), how did you usually get your own cigarettes?	95.0
6	During the past 30 days (one month), what brand of cigarettes did you usually smoke?	97.1
7	How much do you usually pay for a pack of 20 cigarettes?	90.1
8	During the past 30 days (one month), how much do you think you spent on cigarettes?	90.6
12	Where do you usually smoke?	86.9
13	Do you ever have a cigarette or feel like having a cigarette first thing in the morning?	85.3

The internal consistency of attitude-related items was assessed by treating smoking susceptibility (3 items), attitude toward smoking (5 items), and media messages about smoking (7 items) as 3 separate subscales. Cronbach's alpha for scores on these 3 subscales ranged from 0.70 to 0.94, indicating good internal consistency of attitude-related subscales of the Chinese-version GYTS (Table 4).

**Table 4:** Internal consistency (Cronbach's alpha) of the subscales for smoking susceptibility, attitudes toward smoking, and media messages about smoking (*N *= 382)

Subscale	Item number	Item content	Cronbach's α
Smoking susceptibility	15	If one of your best friends offered you a cigarette, would you smoke it?	0.94
	17	At any time during the next 12 months do you think you will smoke a cigarette?	
	18	Do you think you will be smoking cigarettes 5 years from now?	
Attitude toward smoking	20	Do you think boys who smoke cigarettes have more or less friends?	0.70
	21	Do you think girls who smoke cigarettes have more or less friends?	
	22	Does smoking cigarettes help people feel more or less comfortable at celebrations, parties, or in other social gatherings?	
	23	Do you think smoking cigarettes makes boys look more or less attractive?	
	24	Do you think smoking cigarettes makes girls look more or less attractive?	
Media messages about smoking	41	During the past 30 days (one month), how many anti-smoking media messages (e.g., television, radio, billboards, posters, newspapers, magazines, movies) have you seen?	0.71
	42	When you go to sports events, fairs, concerts, community events, or social gatherings, how often do you see anti-smoking messages?	
	43	When you watch TV, videos, or movies, how often do you see actors smoking?	
	45	During the past 30 days (one month), when you watched sports events or other programs on TV how often did you see cigarette brand names?	
	46	During the past 30 days (one month), how many advertisements for cigarettes have you seen on billboards?	
	47	During the past 30 days (one month), how many advertisements or promotions for cigarettes have you seen in newspapers or magazines?	
	48	When you go to sports events, fairs, concerts, or community events, how often do you see advertisements for cigarettes?	

### Test-retest reliability of the Chinese-version GYTS

The test-retest reliability of the Chinese-version GYTS was assessed in 89 students from two randomly selected classes that had completed the questionnaire 2 weeks earlier. The distribution of subscale scores for smoking susceptibility, attitude toward smoking and media messages about smoking were similar at test and retest (Table 5), indicating good reliability of attitude-related subscales of the Chinese-version GYTS.

**Table 5:** Test-retest reliability of scores on attitude-related subscales of the Chinese-version GYST (*n *= 89)

Subscale	Test		Retest		*p*
		
	Mean score	SE	Mean score	SE	
Smoking susceptibility	1.1	0.2	1.2	0.2	0.82
Attitude toward smoking	7.0	0.2	6.9	0.3	0.87
Media messages about smoking	14.8	0.3	14.8	0.3	0.86

## Discussion

The prevalence of tobacco use in Taiwan, as in other countries with Chinese populations, is extremely high compared with western countries. To monitor and control tobacco use in Taiwan, it is crucial to have a reliable and internationally comparable instrument for surveying smoking behavior. The challenge of establishing an international surveillance system is not only to develop a standardized questionnaire but also to deal with language and cultural barriers. Although the GYTS has been established in many countries and translated into several languages such as Spanish, French, and Arabic, no version has been developed by a cross-cultural adaptation process.

The goal of translating a universal survey instrument is to achieve equivalence between the original version and its translated version [13]. That goal is reflected in the Chinese-version GYTS, whose semantic and conceptual equivalence was obtained by carefully executing forward translation, expert review, focus group discussion by students and teachers, and back translation, as recommended for the cross-cultural adaptation process [11,13-15].

Since the original GYTS assesses not only tobacco-related behaviors but also subjective attributes of smoking susceptibility, tobacco-related attitudes, and media literacy, the minimum prerequisite for acceptance of a translated tool is not only the accuracy of its translation but also its validity and reliability [13]. Thus, the content validity and face validity of the Chinese-version GYTS were ensured by an expert committee and focus group discussion. As the nature of item questions varies, response options were scaled as either categorical or ordinal data. Therefore, the internal consistency and stability of this instrument were examined by focusing on the attitude-related subscales of smoking susceptibility, attitudes toward smoking, and media messages about smoking, whose responses have similar scaling systems. Internal consistency for these subscales was estimated by Cronbach's alpha coefficient, the most popular index of reliability for estimating the internal consistency of multiple items on a composite scale [13]. Cronbach's alphas for these subscales were all above 0.70, indicating good internal consistency for items in these subjective domains [16]. The stability of responses was estimated over a 2-week interval, which has been used as a test-retest approach [13]. Similar score distributions on tests taken 2 weeks apart supported the stability of these 3 tobacco attitude-related subscales. In addition to the consistency of responses to items related to subjective attributes, high agreement was found among responses to items related to tobacco-use behaviors, indicating good reliability of the Chinese-version GYTS.

Before these results can be considered in more detail, this study has several limitations that merit attention. First, self-reported smoking behaviors cannot be directly validated because no bioassay is available for physiological evidence of smoking, e.g., serum nicotine. Second, because the data were collected anonymously, coefficients of test-retest reliability could not be estimated; instead we examined the difference in score distributions of 3 subscales between the test and retest. Third, the single focus group of participants selected mainly from the metropolitan area of Taipei may not reflect the cultural diversity of rural areas. Fourth, teachers/staff taking part in the focus group were responsible for teaching health education at their schools, but their relationship with the students was friendlier than traditional Taiwanese teacher-student relationships. We do not know whether this difference would help or hinder students from stating their opinions about tobacco behaviors. Fifth, all twelfth-grade students were excluded in the field test, which may limit generalization of the study findings.

## Conclusion

In the Chinese-version GYTS core questionnaire, 41 items were kept with exactly the same content and format as the original GYTS, making the survey internationally comparable. However, the content of the other 15 items, especially their response categories, were slightly modified to suit current socio-cultural conditions in Taiwan. Our preliminary findings support use of the Chinese-version GYTS for surveillance and research on the prevalence of smoking-related issues in Taiwan. The GYTS was adapted for use in Taiwan by using a cross-cultural adaptation process [11], which includes forward translation, expert committee review, focus group discussion, back translation, and a field test to quantify the results. Our study makes an important contribution to international tobacco control, not only by offering the Chinese version of the GYTS but also a model for translating the English-version GYTS. The Chinese-version GYTS can also serve as a reference for other Chinese communities such as in China, Hong Kong and Macao to plan surveillance and tobacco control programs for teenage smoking behavior.

## Competing interests

The authors declare that they have no competing interests.

## Authors' contributions

PLC participated in designing and administering the study, helped perform statistical analyses, and drafted the manuscript. HYC conceived the study and participated in its design. YHC participated in and supervised the design, administered the study, and performed statistical analyses. All authors read and approved the final manuscript.

## Pre-publication history

The pre-publication history for this paper can be accessed here:



## Supplementary Material

Additional file 1 Final Chinese version of Global Youth Tobacco Survey. Chinese-version GYTS distributed to Taiwanese youth (in Mandarin). Click here for file
