# Supplementary material for: Chinese version of the Global Youth Tobacco Survey: cross-cultural instrument adaptation
Source: BMC Public Health. 2008 Apr 30;8:144. doi: 10.1186/1471-2458-8-144 (PMC2390538; doi:10.1186/1471-2458-8-144)
Supplement: Additional file 1 — Final Chinese version of Global Youth Tobacco Survey. Chinese-version GYTS distributed to Taiwanese youth (in Mandarin). [file 1471-2458-8-144-S1.doc]

Final Chinese version of Global Youth Tobacco Survey

1. 請問您是否嘗試過吸菸，即使只吸一、兩口？
2. 是
3. 否
4. 您第一次吸菸是幾歲的時候？
5. 我連一口菸都未曾嘗試
6. 7歲或7歲以前
7. 8~9歲
8. 10~11歲
9. 12~13歲
10. 14~15歲
11. 16歲(含)以後
12. 到目前為止，您是否曾吸菸超過100支？
    1. 是
13. 否
14. 過去30天（一個月）內，您一共吸菸多少天呢？
    - - 1. 這段時間，我沒有吸菸
        2. 1或2天
        3. 3到5天
        4. 6至9天
        5. 10到19天
        6. 20到29天
        7. 每天都有
15. 過去30天（一個月）內，您有吸菸的日子中，平均每天吸幾根香菸？
    1. 這段時間，我沒有吸菸
    2. 每天少於1根香菸
    3. 每天1根香菸
    4. 每天2-5根香菸
    5. 每天6-10根香菸
    6. 每天11-20根香菸
    7. 每天20根香菸以上
16. 過去30天（一個月）內，您通常如何取得香菸？
    - 1. 這段期間，我沒有吸菸
      2. 我從商店買的，如：便利商店、超商、免稅商店或加油站
      3. 我付錢，請別人幫我買
      4. 我向別人借的
      5. 我偷來的
      6. 年紀較大的人給我香菸
      7. 其他
17. 過去30天（一個月）內，您通常吸什麼牌子的菸呢？
18. 這段期間，我沒有吸菸
19. 沒有固定
20. 七星(Mild Seven)
21. 長壽(Long Life)
22. 大衛杜夫(Davidoff)
23. 萬寶路(Marlboro)
24. 維珍妮(Virginia)
25. 其他牌子
26. 過去30天（一個月）內，您通常花費多少錢買一包20支的菸？
27. 這段期間，我沒有吸菸
28. 我有吸菸但我沒有買菸，或我沒有買整包的菸
29. 35元以下
30. 36~45元
31. 46~55元
32. 56元以上
33. 過去30天（一個月）內，您一共花了多少錢買菸呢？
34. 這段期間，我沒有吸菸
35. 我有吸菸但我沒有買菸
36. 200元以下(約4包)
37. 200~399元(約8包)
38. 400~599元(約12包)
39. 600~799元(約16包)
40. 800元以上(約20包)
41. 通常一個月（30天）裡，您有多少零用錢呢（包含打工收入）？
42. 我沒有任何零用錢
43. 少於500元
44. 500~1499元
45. 1500~2499元
46. 2500~3499元
47. 3500~4499元
48. 4500元以上
49. 過去30天（一個月）內，有人因您的年齡而拒絕賣菸給您嗎？
50. 過去30天內我沒有去買菸
51. 有的，過去30天內有人因我的年齡而拒絕賣菸給我
52. 沒有，我的年齡並沒有影響我買菸
53. 過去30天（一個月）內，除了香菸以外，您是否曾經使用其他的香菸製品（例如嚼菸絲、抽雪茄、煙斗）？
    - 1. 有
      2. 沒有
54. 您最常在那裡吸菸？
    - - 1. 我從未吸菸
        2. 在家裡
        3. 在學校
        4. 在工作的地方
        5. 在朋友的住處
        6. 在社交場合（如朋友聚會、親友婚宴等活動）
        7. 在公共場所（如公園、百貨公司、街上）
        8. 其他
55. 您是否有在早上起床後馬上就吸菸或想要吸根菸的情形？
    - - 1. 我從未吸菸
        2. 我很久沒有吸菸
        3. 沒有，我沒有在早上起床後馬上就吸菸或想要吸菸
        4. 有，我有時會在早上起床後馬上就吸菸或想要吸菸
        5. 有，我總是會在早上起床後馬上就吸菸或想要吸菸
56. 您的父母目前有沒有吸菸？
57. 父母親都沒有吸菸
58. 父母親都有吸菸
59. 只有父親吸菸
60. 只有母親吸菸
61. 我不知道
62. 如果您的好朋友給您1支菸，您會吸它嗎？
63. 一定不會吸
64. 可能不會吸
65. 可能會吸
66. 一定會吸
67. 您的家人是否曾與您談過吸菸的害處？
68. 有，曾經談過
69. 沒有談過
70. 在未來的一年內，您想您會不會吸菸？
    1. 一定不會吸
    2. 可能不會吸
    3. 可能會吸
    4. 一定會吸
71. 在未來的五年內，您想您會不會吸菸？
    - 1. 一定不會吸
      2. 可能不會吸
      3. 可能會吸
      4. 一定會吸
72. 一旦開始吸菸，您認為是不是會很難戒掉？
    1. 一定不會很難戒掉
    2. 可能不會很難戒掉
    3. 可能會很難戒掉
    4. 一定會很難戒掉
73. 會吸菸的男孩，您認為會有比較多或比較少的朋友嗎？
74. 會有比較多朋友
75. 會有比較少朋友
76. 沒有影響
77. 會吸菸的女孩，您認為會有比較多或比較少的朋友嗎？
    1. 會有比較多朋友
    2. 會有比較少朋友
    3. 沒有影響
78. 吸菸是否會讓人在慶典、舞會或其他社交場合覺得比較自在？
79. 比較自在
80. 比較不自在
81. 沒有影響
82. 您認為吸菸會讓男孩看起來更有吸引力嗎？
83. 會更有吸引力
84. 會更沒有吸引力
85. 沒有影響
86. 您認為吸菸會讓女孩看起來更有吸引力嗎？
87. 會更有吸引力
88. 會更沒有吸引力
89. 沒有影響
90. 您認為吸菸會讓您的體重增加或減輕？
91. 會增加體重
92. 會減輕體重
93. 沒有差別
94. 您認為吸菸會損害您的健康嗎？
95. 一定不會
96. 可能不會
97. 可能會
98. 一定會
99. 您的好朋友吸不吸菸？
100. 都沒有吸菸
101. 有一些有吸菸
102. 大部份有吸菸
103. 全部都有吸菸
104. 當您看到一個男人在吸菸，您會怎麼想他？
105. 缺乏自信
106. 愚蠢
107. 隨便
108. 失敗者
109. 成功者
110. 有智慧的
111. 粗曠的
112. 帥氣的
113. 當您看到一個女人在吸菸，您會怎麼想她？
114. 缺乏自信
115. 愚蠢
116. 隨便
117. 失敗者
118. 成功者
119. 有智慧的
120. 成熟的
121. 優雅的
122. 您認為吸菸1～2年後就戒掉，對身體會不會有危害？
123. 一定不會有危害
124. 可能不會有危害
125. 可能會有危害
126. 一定會有危害
127. 您認為吸二手菸會危害您的健康嗎？
128. 一定不會
129. 可能不會
130. 可能會
131. 一定會
132. 在過去七天內，您在家時，有人在您的面前吸菸的天數有幾天？
133. 0 天
134. 1~2天
135. 3~4天
136. 5~6天
137. 7天
138. 在過去七天內，您在家以外的地方時，有人在您的面前吸菸的天數有幾天？
139. 0 天
140. 1~2天
141. 3~4天
142. 5~6天
143. 7天
144. 您贊不贊成公共場所禁菸？（如餐廳、公共汽車、火車、學校、遊戲場、健身房、運動場、舞廳）
145. 贊成
146. 不贊成
147. 您現在想不想戒菸？
148. 我連一口菸都未曾嘗試
149. 我吸菸未超過100支
150. 我曾經吸菸，但是現在已經不吸菸了
151. 想戒菸
152. 不想戒菸
153. 在過去一年裡，您是否曾試過戒菸？
154. 我連一口菸都未曾嘗試
155. 我吸菸未超過100支
156. 我戒菸已經超過一年了
157. 曾經試過戒菸
158. 沒有試過戒菸
159. 您戒菸多久了？
160. 我連一口菸都未曾嘗試
161. 我吸菸未超過100支
162. 我從未戒菸
163. 1~3個月
164. 4~11個月
165. 一年
166. 兩年
167. 三年或更久
168. 您決定戒菸的最主要原因是什麼？
169. 我連一口菸都未曾嘗試
170. 我吸菸未超過100支
171. 我從未戒菸
172. 改善我的健康
173. 省錢
174. 我的家人不喜歡我吸菸
175. 我的朋友不喜歡我吸菸
176. 其他
177. 您認為您想戒菸就能戒掉嗎？
178. 我連一口菸都未曾嘗試
179. 我吸菸未超過100支
180. 我已經戒菸了
181. 我想戒菸就能戒掉
182. 我想戒菸不見得能戒掉
183. 是否曾經有人幫助或勸告您戒菸？
184. 我連一口菸都未曾嘗試
185. 我吸菸未超過100支
186. 曾接受過戒菸課程或專業人員的幫助
187. 曾接受過朋友的幫助
188. 曾接受過家人的幫助
189. 曾接受過戒菸課程或專業人員，以及朋友或家人的幫助
190. 沒有
191. 在過去30天（一個月）內，您看過或聽過多少有關反菸的媒體資訊（如電視、收音機、廣告看板、海報、報紙、雜誌、電影）？
192. 很多
193. 有一些
194. 沒有
195. 當您參加運動會、園遊會、音樂會、社區活動或社交聚會時，您是否常看到有關反菸的訊息？
196. 我從未參加過運動會、園遊會、音樂會、社區活動或社交聚會
197. 經常會看到
198. 有時會看到
199. 從來都沒有看到
200. 當您觀賞電視、錄影帶或電影時，您是否常看到演員吸菸？
201. 我從未看電視、錄影帶或電影
202. 經常會看到
203. 有時會看到
204. 從來都沒有看到
205. 您有沒有任何東西上面印著香菸品牌的標誌（如Ｔ恤、筆、背包等等）？
206. 有
207. 沒有
208. 在過去30天（一個月）內，當您觀賞電視上的體育活動或其他節目時，是否常看到香菸品牌的廣告？
     1. 我從未看電視
     2. 經常會看到
     3. 有時會看到
     4. 從來都沒有看到
209. 在過去30天（一個月）內，您看過多少香菸廣告的看板？
210. 很多
211. 有一些
212. 沒有
213. 在過去30天（一個月）內，您看過多少報紙或雜誌上有香菸廣告？
214. 很多
215. 有一些
216. 沒有
217. 當您參加運動會、園遊會、音樂會或社區活動時，您是否常看到香菸廣告？
     1. 我從未參加過運動會、園遊會、音樂會或社區活動
     2. 經常會看到
     3. 有時會看到
     4. 從來都沒有看到
218. 是否曾有香菸公司的人提供您免費的香菸？
     - 1. 曾
       2. 不曾
219. 在過去一年中，您是否曾經上過有關吸菸危害健康的課？
220. 有
221. 沒有
222. 不確定
223. 在過去一年中，您是否曾經在課堂上討論過「和您同年齡的人為什麼會吸菸的原因」？
224. 有
225. 沒有
226. 不確定
227. 在過去一年中，您是否曾經上過吸菸對人體的影響，例如使牙齒變黃、形成皺紋、讓人聞起來味道不好的課？
228. 有
229. 沒有
230. 不確定
231. 上一次在課堂上曾討論吸菸與健康的問題是多久以前？
232. 從來沒有
233. 這一學期
234. 上一學期
235. 兩個學期以前
236. 三個學期以前
237. 二年或更久以前
238. 請問您的年齡是：
239. 11歲(含)以下
240. 12歲
241. 13歲
242. 14歲
243. 15歲
244. 16歲
245. 17歲(含)以上
246. 請問您的性別是：
247. 男
248. 女
249. 請問您目前就讀的年級是：
250. 國中一年級 (高中一年級)
251. 國中二年級 (高中二年級)
252. 國中三年級 (高中三年級)
